# Evaluation of Oral and Dental Health in Patients with Rheumatoid Arthritis: A Prospective Case-control Study: Oral and Dental Health and Rheumatoid Arthritis

**DOI:** 10.31661/gmj.v13iSP1.3516

**Published:** 2024-12-31

**Authors:** Naghmeh Salesi, Fatemeh Rashidi Meibodi, Hajar Shekarchizadeh, Erfan Mohammadi, Mansour Salesi

**Affiliations:** ^1^ Isfahan (Khorasgan) Branch, Islamic Azad University, Isfahan, Iran; ^2^ Department of Oral and Maxillofacial Medicine, School of Dentistry, Isfahan (Khorasgan) Branch, Islamic Azad University, Isfahan, Iran; ^3^ Department of Community Oral Health, School of Dentistry, Isfahan (Khorasgan) Branch, Islamic Azad University, Isfahan, Iran; ^4^ Community Health Research Center, Isfahan (Khorasgan) Branch, Islamic Azad University, Isfahan, Iran; ^5^ Department of Rheumatology, School of Medicine, Isfahan University of Medical Sciences, Isfahan, Iran

**Keywords:** Oral Health, Rheumatoid Arthritis, Disease Activity Score

## Abstract

**Background:**

Rheumatoid Arthritis (RA) is an autoimmune disease characterized by chronic inflammation. The present study aims to investigate the oral and dental health including periodontal status and decayed, missing, and filled teeth (DMFT) index in RA patients, who were divided into three groups by disease activity score-28 (DAS-28) classification: silent, active, and semi-active.

**Materials and Methods:**

This case-control study was conducted during January to December 2022. There were 54 patients in the case group who were divided into three groups based on the DAS index: mild, moderate, and severe. Fifty-four healthy individuals were in the control group. DMFT and DAS-28 indices were used to evaluate the oral health of patients. The evaluation of these indexes was done based on standard questionnaires. The chi-square, Mann-Whitney, and Kruskal-Wallis tests were used to analyze data based on variable type. Analysis was performed at the 95% confidence interval using SPSS software version 24.

**Results:**

Patients with severe activity showed more pocket depth and clinical attachment lost (CAL) (P0.001). No statistical significance was observed in the comparison of DMFT index between the healthy group and patients with mild, moderate, and severe disease activity (P=0.339); patients had lost more teeth than the control group (P0.001).

**Conclusion:**

Based on the results, it seems that RA patients with severe activity have a worse condition than the control group. However, according to the result, there was not significant difference in the DMFT index and bleeding on probing among patients with three different severities and healthy individuals.

## Introduction

Rheumatoid arthritis (RA) is an autoimmune disease that causes inflammation in many parts of body. The inflammatory process usually involves the joints of the hands and feet, which if not treated may cause serious damage to the joints and disrupts the function of joints; ultimately it causes disability. RA occurs at a ratio of three to one in women compared to men [1][2].

RA treatment primarily is managed with painkillers, non-steroidal anti-inflammatory drugs (NSAIDs), biological and non-biological DMARDs, and physical and surgical treatments that reduce the symptoms and swelling of the affected joints. Dry mouth is one of the common manifestations of RA patients, so, it is said that almost half of these patients are involved with this condition. On average, these patients have a 15-30% decrease in saliva secretion, which is mostly related to the parotid gland. Decreased secretion of salivary glands depends on the severity and duration of disease [3][4].

Periodontitis includes a range of diseases that affect the tissues around the teeth. It can cause bone destruction around the teeth. Among the main characteristics of periodontitis are bleeding gums, bone loss, periodontal pocket formation, and tissue inflammation [5]. Periodontitis is a chronic inflammatory condition in periodontal tissues (gums, periodontal ligaments, and alveolar bone) that occurs due to immune response of the host to the microbial biofilm on the tooth’s surface. The immune reaction destroys periodontal tissues around the teeth [6][7][8][9].

In recent studies, the correlation between RA and periodontitis has been proven; this issue is due to numerous similarities in pathological and immunological features, including an increase in immune cells including monocytes, neutrophils, B and T lymphocytes, secretion of inflammatory cytokine which increases the secretion of inflammatory mediators by immune cells [10][11][12][13]. In Mercado’s study [14].It has been stated that the index of bleeding from the gums and the amount of plaque in the control and the patient groups were not significantly different; the patients affected by RA showed greater pocket depth than the control group.

Dry mouth is one of the side effects observed in RA patients. This complication can cause many problems in patients. There are different indexes used to check oral health. It is known that the DMFT index is used as a standard index to check the state of oral and dental health. So far, very few studies have been conducted concerning oral and dental hygiene in RA patients. The present study aims to investigate the oral and dental health including periodontal status and DMFT index in RA patients, who were divided into three groups by DAS-28 classification: silent, active, and semi-active.

## Materials and Methods

**Table:** Table1. Description and Demographical Information of the Two Groups

		Disease Severity			Healthy group		Kruskal-wallis H	p-value
		Mild N (%)	Moderate N (%)	Severe N (%)				
Gender	Male	9(50)	9(50)	9(50)	27(50)	00		1.00
	Female	9(50)	9(50)	9(50)	27(50)			
Age		56.11 ±7.52	48.67± 12.87	49.28±9.96	47.91 ±8.58		21.44	0.34
								c*
Duration of disease		9.56±7.63	11.56±10.77	11.58±8.41			91.99	<0.001
								a*
Sulfasalazine	Yes	2(11.1)	3(16.7)	4(22.2)		0.80		0.67
	No	16(88.9)	15(83.3)	14(77.8)				b*
Methotrexate	No	4(22.2)	5(27.8)	4(22.2)		0.20		0.90
	Yes	!ww1hf	13(72.2)	14(77.8)				b*
NSAID	Yes	9(50)	5(27.8)	7(38.9)		1.87		0.39
	No	9(50)	13(72.2)	11(61.1)				a*
Prednisolone	Yes	15(83.3)	16(83.3)	18(100)		3.08		0.21
	No	3(16.7)	2(16.7)	0(0)				b*
Biologic drugs	Yes	7(38.9)	2(11.1)	3(16.7)		4.50		0.10
	No	11(61.1)	16(88.9)	15(83.3)				b*
Hydroxychloroquine	Yes	8(44.4)	12(66.7)	11(61.1)		1.96		0.37
	No	10(55.6)	6(33.3)	7(38.9)				b*

Calculated based on (a) fisher exact and (b) chi-squared test (c) one-way ANOVA test

This case-control study was conducted in Azad university of Isfahan branch (Ethical code: IR.IAU.KHUISF.REC.1401.039) from January to December 2022. The RA patients who had been diagnosed for at least two years with medical records and were under treatment were included in the study; the age range of the case group was 30-60 years. The gender of the patients in both groups was the same. However, the age of the participants was chosen as close to each other as possible based on the limited availability of RA patients.

### Inclusion and Exclusion

The patient age range of the case group was 30-60 years. Patients with diseases such as diabetes mellitus, osteoporosis, cardiovascular disease, malignancy, kidney failure, pregnant patients, who had a history of recent periodontal surgery, completely edentulous patients, and individuals with a history of taking anticoagulant drugs were excluded from the study.

### Disease Activity Score (DAS)

The disease activity assessment index or DAS is specific to RA patients. Various factors are evaluated in this index, including laboratory tests (ESR), the patient’s feedback about his disease, and the number of swollen and sensitive joints out of 28 joints [15].

### Clinical Examination

The DAS index is a clinical examination evaluating the BOP and PPD. For this purpose, the CPI index was used. In the CPI index the dental system is divided into 6 sextants: 1. Posterior right maxilla (from tooth 17- 14), 2. Anterior maxilla (13-23), 3. Posterior left maxilla (24-27), 4. Posterior left mandible (34-37), 5. Anterior mandible (33-43), and 6. Posterior right mandible (44-47).

To check the CAL the dentition is divided into 6 quadrants 1. As mentioned previously, the codes 0: no analysis and loss of adhesion, 1: 1mm>CAL>2mm, 2: 3mm>CAL>4mm, and 3: CAL>5mm are used for the report. Then, the highest score recorded for each quadrant will be averaged.

To evaluate the BOP, the periodontal probe is gently moved laterally in the depth of the pocket, and then bleeding from the gums is checked. Sometimes immediately after probing and sometimes after a few seconds bleeding occurs; after completing the probing, 30-60 seconds should be spent and then bleeding evaluated.

### DMFT Index

A table was prepared to check the DMFT index, and all teeth except wisdom teeth were examined; decayed teeth scored with code 1, restored teeth that need to be replaced or have secondary decay with code 2, restored teeth with code 3, missing teeth with code 4, and finally any cover, implant, bridge, and veneer with code 5. Finally, the total number of decayed (DT), filled (FT), and missing (MT) teeth was calculated and recorded. Finally, all these numbers were added together and the DMFT index was obtained. Based on the obtained data necessary analysis was done. It should be mentioned that the detection of interdental caries in the case group was done by the trans illumination test and the dental explorers were used to evaluate the caries of the smooth surfaces (Class v) and the occlusal surface; to detect the caries of the control group, transillumination tests and the dental explorers were used.

### Statistical Analysis

The analysis was done on the two descriptive and inferential levels. At the descriptive level, the frequency distribution tables and the mean and standard deviation indices were used to describe the situation of the sample in each of the two groups. At the inferential level to control the presumption of data normality, the Shapiro-Wilk test was used; if this assumption was established, the test Kruskal-Wallis and Mann-Whitney tests were performed. To compare the quantitative variables between patients with different severity of the disease chi-square test was used. Analysis was performed at the 95% confidence interval using SPSS software version 24.

### Ethical Approval

All the procedures performed in the studies involving human participants were following the ethical standards of the local ethics committee of Isfahan University of Medical Science (IR.IAU.KHUISF.REC.1401.039), as well as the 1964 Helsinki declaration.

## Results

**Table:** Table2. Comparison of Habits and Behaviours affecting Oral and Dental Health between the Two Groups

		**Case group** **N (%)**	**Control group** **N (%)**	**χ** **2**	**p-value**
**Teeth brushing **	Favourable	33 (61.1)	22 (40.7)	0.039	0.844
	unfavourable	22 (38.9)	32 (59.3)		a*
**Use toothpaste **	favourable	46(85.2)	53(98.1)	-	0.031
	unfavourable	8(14.8)	1(1.9)		b*
**Use dental floss **	favourable	16(29.6)	12(22.2)	0.278	0.380
	unfavourable	38 (70.4)	42(77.8)		a*
**Last visit to the dentist **	favourable	25(46.3)	12(22.2)	5.948	0.008
	unfavourable	29(53.7)	42(77.8)		a*
**Eating sweet snacks **	favourable	43(79.6)	37(68.5)	1.736	0.188
	unfavourable	11(20.4)	17(31.5)		a*
**smoking**	favourable	51(94.4)	46(87)	1.763	0.184
	unfavourable	3(5.6)	7(13)		a*

Calculated based on (b) Fisher’s exact and (a) chi-squared test

**Table:** Table3. Comparison of DMFT Index of Research Units based on Disease Severity

		Mean ±SD	P-value
		Mild	18.89 ±6.34		
DMFT index	moderate	18.83 ±5.54	0.33
	Severe	16.94±6.1	a*
	Healthy	16.78±4.03	
DMFT index	Case	18.22 ±5.96	0.195
	Control	16.78 ±4.03	b*

Calculated based on Kruskal-Wallis test (a) and Mann-Whitney test (b)

### Demographical Information

Based on the analyses, the results showed that the gender of the individuals in both groups was the same. Half of the individuals were male and the other 50% were female (P=1). The mean age in the control group was 47.91 ± 8.58 and in the patient group based on mild, moderate, and severe was 56.11 ± 7.52, 48.67 ± 12.87, and 49.28 ± 9.96, respectively (P=1). The mean duration of disease in patients with severe disease was significantly longer than mild and moderate cases (P<0.001). In addition, there was no significant relationship between the patients in terms of medication history (P>0.05, Table-1).

### Evaluation of Habits and Behaviours between the Two Groups

In the comparison between the two groups in terms of habits and behaviours affecting oral and dental health, a significant difference was observed between the two groups in using toothpaste and going to the dentist. According to the result of the Chi-square test, the favourable behaviour in using toothpaste among healthy people was significantly higher than patients (P=0.031). Also, in terms of going to the dentist as a favourable behaviour, it was significantly higher among patients compared to healthy individuals (P=0.008).

Other habits include brushing teeth (P=0.844), using dental floss (P=0.38), eating sweet comestibles and snacks, (P=0.188), and smoking (P=0.184, Table-2).

### Evaluation of DMFT Index of Research Units based on Disease Severity

**Figure-1:**
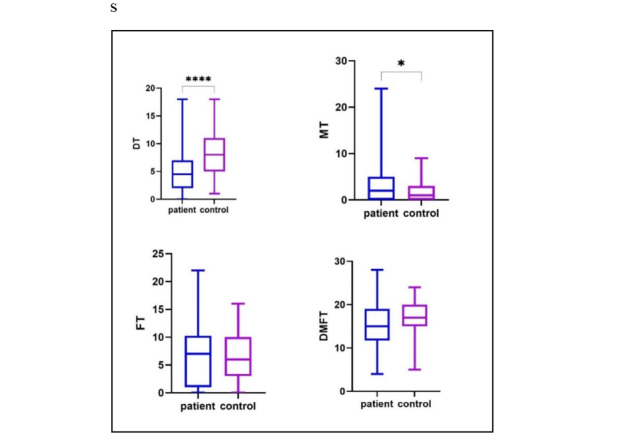
Figure 1. Comparison of the DMFT index between the two groups. In each group, the middle line is equal to the median value of observations. The mean of FT and MT was higher in RA patients however, this difference was statistically significant only for MT. On the other hand, the mean values of DMFT and DT were higher in the control group compared to the patient group.

Mann-Whitney test was used to compare the DMFT index between the two groups. Based on the results of this test, there was no significant difference between the groups (P=0.195, Table-3). Of course, the Mann-Whitney test showed that the average number of decayed teeth in healthy people (8.33 ± 3.77) was higher than other groups (6.63 ± 4.93), and the average number of missing teeth in healthy people (2.29 ± 1.85) was lower than the patients (5.39 ± 5.86). The average number of filled teeth between the RA (6.20 ± 5.61) and healthy (6.59 ± 4.09) groups was similar (Figure-1).

### Evaluation of CPI Index in Research Units based on Disease Severity

The result of the Chi-square test showed a significant difference comparing of CPI index in research units based on disease severity; it was significantly different in patients with severe and mild and moderate disease (P<0.001, Table-4).

### Evaluation of the BOP Index in Research Units based on Disease Severity

The frequency of patients with the BOP index was higher compared to patients without it. According to the chi-square test, there was no significant difference between the BOP index in research units based on disease severity (P=0.526, Table-5).

### Evaluation of CAL Index in Research Units based on Disease Severity

The result of the Chi-square test showed a significant difference between healthy and RA cases (mild, moderate, and severe) in terms of CAL index value (P<0.001, Table-6).

## Discussion

**Table:** Table4. Comparison of CPI Index in Research Units based on Disease Severity

			**Disease severity**		**Healthy** **N (%)**		
		Mild N (%)	Moderate N (%)	Sever N (%)		χ2	p-value
	Healthy	0	3(16.7)	0	9(16.7)		
	Bleeding	0	1(5.6)	1(5.6)	11(20.4)		
**CPI index**	Calculus	5(7.8)	7(38.9)	3(16.7)	19(35.2)	22.147	<0.001
	Pocket 4-5mm	9(50)	4(22.2)	7(38.9)	10(18.5)		
	Pocket >6mm	1(5.6)	3(16.7)	8(44.4)	5(9.3)		

**Table:** Table5. Comparison of BOP Index in Research Units based on Disease Severity

			**Case group**		**Healthy**		
		Mild	Moderate	Severe	N (%)	χ2	p-value
		N (%)	N (%)	N (%)			
**BOP index**	Have	14(77.8)	12(66.7)	14(77.8)	34(63)	2.233	0.526
	Don’t have	4(22.2)	6(33.3)	4(22.2)	20(37)		

Calculated based on the Chi-square test

The results of this study about behaviours affecting oral and dental health showed that visiting the dentist was significantly higher in patients with RA compared to another group. Probably it means that the patient is always worried about the disease becoming active and out of control after the smallest problem; in other words, it has become intertwined with the patient’s life and has changed their lifestyle, and patients should have regular medical follow-ups; so that, the treatment process would be more successful [16].

There was no significant difference in other parameters such as brushing teeth, the amount of flossing and the snacks consumed in the patient and the control group. It was similar to the result of Shim et al. Also, in the present study, there was no significant difference between the use of toothbrushes and smoking between the case and control groups [17].

In the current study, the research results indicated that there was a significant difference between healthy and sick people with three degrees of mild, moderate, and severe in terms of the CPI index value; people with severe and mild disease had significantly higher mean of the CPI index. Also, the result showed that the pocket depth was higher in severe and mild group patients than the healthy people. Another comparison between patients and healthy people revealed that sick people had a significantly greater pocket depth.

In the study of Qadir et al, it was shown that the average CPI index was higher in RA patients compared to the healthy group [18]. Also, in another study, it was found that the average CPI index increment was associated with the increase in symptoms of RA patients [19].

In the case of the CAL index, the results were similar to the CPI; in this index, both patients with mild and severe severity showed more CAL than the control group; in the comparison between the healthy and RA groups, the patients showed significantly more CAL than the healthy people. It seems that higher CAL and pocket depth in mild RA patients in the present study are related to the higher age average of these patients compared to another group. Some inflammatory mediators such as cytokines, HLA-DR antigens, hormones, and even specific periodontal pathogens such as Porphyromona gingivalis can be involved in the relationship between periodontitis and RA [20][21].

Recent studies conducted by De Pablo [22], Jung [23], and Detert [24] showed that these patients are more exposed to periodontitis than other people. Although there are studies conducted by Laurell [25], Yavuzyilmaz [26], and Helminen [27], which did not determine any relationships between periodontitis and RA. In Mercado’s study [14] has been stated that the index of bleeding from the gums and the amount of plaque in the control and the patient groups were not significantly different; the patients affected by RA showed greater pocket depth than the control group. Studies about RA and periodontitis [28][29] both share a common biomedical mechanism of bone destruction caused by inflammatory cytokines. It should be noted that medications used to treat RA may affect the risk of periodontitis, which may confound the observed association between RA and periodontal disease. Medications taken by people with RA (such as glucocorticoids) are a known cause of osteoporosis and systemic demineralization, which may predispose the patient to further bone loss and periodontitis.

The BOP index did not show any significant differences between healthy and sick people with any severities, which is in line with the results of the Mercado study [14]. Comparing the DMFT index in healthy people versus RA cases showed no significant difference.

In the general comparison between sick and healthy people, the observations indicated that the number of decayed teeth was more in the control group, but the number of missing teeth was significantly more in the patients. There was no correlation between the filled teeth and the DMFT index between the healthy and RA groups.

In the study of Rovas et al, it was also shown that in line with the present study, there was no correlation between the BOP index in RA patients with oral disorders and healthy people [30]. In a meta-analysis study, it was shown that the average BOP was higher in RA patients compared to normal subjects, and the increment was associated with the incidence of inflammation in patients [31]. Also, Jayatilake et al. showed that the increase in BOP in RA patients was associated with oral bleeding and periodontal disease [32].

It is necessary to have an overview of the definition of oral health in these patients. In general, health refers to a state of complete physical, mental, and social well-being. The level of health is affected by the state of objective and mental health [14][28]. According to this definition, RA patients do not have an ideal level of health due to being involved with a chronic disease. In general, patients with RA may not be able to perform oral and dental hygiene properly due to limited movement of wrist and finger joints. This result is in line with Lgervall’s study [29], in which it is mentioned that people with RA lose more teeth during their lifetime. It is also consistent with Silvestre’s study [33], which states that the DMFT index was not significantly different between the two groups of case and control. The average DMFT index in the present study was relatively similar to the average DMFT in the study of Parvai et al. in 2020 [33].

According to a recent study conducted by Shim et al [17] in 2022, observations indicated that there was a relationship between poor oral health and RA in the diabetic group. It is consistent with a previous study by Huang [34], that reported poor oral health due to tooth decay or periodontitis in adults with diabetes.Only RA patients participated in this study. It is better to investigate other rheumatology patients and compare them in future studies. In addition, the relationship between oral and dental health should be investigated in patients with their type of treatment.

## Conclusion

**Table:** Table6. Comparison of CAL Index in Research Units based on Disease Severity

			**Disease severity**		**Healthy** **N (%)**		
		Mild	Moderate	Sever		χ2	p-value
		N (%)	N (%)	N (%)			
	Normal	5(27.8)	11(61.1)	4(22.2)	37(68.5)		
	Mild	7(38.9)	3(16.7)	7(38.9)	11(20.4)		
**CAL** **index**	Moderate	2(11.1)	4(22.2)	3(16.7)	5(9.3)	16.700	<0.001
	Severe	4(22.2)	0	4(22.2)	1(1.9)		
				

Calculated based on the Kruskal-Wallis test

It seems that RA patients have more serious periodontal conditions than the control group; so, this serious situation is more tangible in patients with severe disease activity. RA patients need to undergo a dental examination that can be recommended by rheumatologists. These people should undergo periodontal evaluation and examination, because there may be a greater risk of periodontitis. Both diseases can overshadow people’s quality of life to a great extent. An important factor to consider is the extent to which the patient’s quality of life is affected by RA and PD.

Furthermore, it seems that a joint approach between rheumatologists and dentists for the management of RA is necessary to determine whether routine periodontal treatments can modulate disease activity by reducing cytokines and pro-inflammatory factors. However, the role of different AS-related parameters, periodontal health conditions, and their relationship seems unclear.

## Conflict of Interest

The authors declare that they have no conflict of interest.
